# Modeling multiple sclerosis using mobile and wearable sensor data

**DOI:** 10.1038/s41746-024-01025-8

**Published:** 2024-03-11

**Authors:** Shkurta Gashi, Pietro Oldrati, Max Moebus, Marc Hilty, Liliana Barrios, Firat Ozdemir, PHRT Consortium, Veronika Kana, Andreas Lutterotti, Gunnar Rätsch, Christian Holz

**Affiliations:** 1 Department of Computer Science, ETH Zürich, Zürich, Switzerland; 2 ETH AI Center, ETH Zürich, Zürich, Switzerland; 3 Institute for Implementation Science in Health Care, University of Zürich, Zürich, Switzerland; 4 Department of Neurology, University Hospital Zürich, Zürich, Switzerland; 5 Swiss Data Science Center, ETH Zürich & EPFL, Zürich, Switzerland

**Keywords:** Multiple sclerosis, Diagnostic markers

## Abstract

Multiple sclerosis (MS) is a neurological disease of the central nervous system that is the leading cause of non-traumatic disability in young adults. Clinical laboratory tests and neuroimaging studies are the standard methods to diagnose and monitor MS. However, due to infrequent clinic visits, it is fundamental to identify remote and frequent approaches for monitoring MS, which enable timely diagnosis, early access to treatment, and slowing down disease progression. In this work, we investigate the most *reliable*, *clinically useful*, and *available* features derived from mobile and wearable devices as well as their ability to distinguish people with MS (PwMS) from healthy controls, recognize MS disability and fatigue levels. To this end, we formalize clinical knowledge and derive behavioral markers to characterize MS. We evaluate our approach on a dataset we collected from 55 PwMS and 24 healthy controls for a total of 489 days conducted in free-living conditions. The dataset contains *wearable sensor data* – e.g., heart rate – collected using an arm-worn device, *smartphone data* – e.g., phone locks – collected through a mobile application, *patient health records* – e.g., MS type – obtained from the hospital, and *self-reports* – e.g., fatigue level – collected using validated questionnaires administered via the mobile application. Our results demonstrate the feasibility of using features derived from mobile and wearable sensors to monitor MS. Our findings open up opportunities for continuous monitoring of MS in free-living conditions and can be used to evaluate and guide the effectiveness of treatments, manage the disease, and identify participants for clinical trials.

## Introduction

Multiple sclerosis (MS) is a chronic neurological disease that affects the central nervous system (CNS), which was discovered by Jean-Martin Charcot in 1868^1^. Over 2.8 million people globally have been diagnosed with MS^2^. It can affect several parts of the CNS leading to a wide range of symptoms, including, pain, mood changes, vision and movement problems, speech difficulties, and balance impairment. MS is a chronic disease, with symptoms often worsening over time. The most occurring and troubling symptom of MS is *fatigue*, which refers to the “*subjective sensations of weariness, increasing sense of effort, mismatch between effort expended and actual performance, or exhaustion*”^3–5^. Recurring fatigue leads to low productivity, sick leave, and work disability^6,7^. It interferes with an individual’s daily activities and reduces the quality of life^3,8^. Considering that currently no cure for MS exists, it is essential to provide treatments to reduce the impact of such symptoms.

To monitor the disease and its symptoms, clinicians largely rely on magnetic resonance imaging (MRI)^9^, clinical rating scales, e.g., *Expanded Disability Status Scale (EDSS)*^10^ – that assess the disease disability level – and validated self-reports, e.g., *Fatigue Scale for Motor and Cognitive function (FSMC)*^11^ or *Visual Analog Scale (VAS)*^12^ – which measure motor and cognitive fatigue. While MRI and clinical rating scales are effective and adequate to approximate a patient’s status at the time of the clinical visit, they can only be performed by trained physicians at clinical centers. In addition, they require frequent MRI scanning and doctor visits to reflect longitudinal changes of MS impairment, which is cumbersome and time-consuming. Self-reports could in principle be deployed for longitudinal monitoring of MS. However, it is difficult to maintain users’ adherence to them over time. Additionally, they are unreliable due to recall and social biases^13^. Due to the aforementioned reasons, there is a need to explore novel techniques to help patients and clinicians monitor MS in natural environments.

Thanks to advancements in mobile and wearable technology, continuous and unobtrusive monitoring of several health aspects has become possible (e.g., sleep^14^, physical activity^15^, and cognitive impairment^16^). These devices, equipped with a wide range of sensors, can measure behavioral and physiological parameters, offering new opportunities for continuous disease monitoring in natural settings. A recent study shows the evolution of the use of digital health technologies in neurology trials, including MS^17^. Computing systems able to model different aspects of MS would help patients and clinicians to effectively and efficiently understand, monitor, and manage the disease. For instance, continuous monitoring of physiological and behavioral signals of PwMS in free-living conditions would allow clinicians to maintain a history of events and behaviors preceding the transient worsening of neurological symptoms. To develop such systems, it is first necessary to understand the *reliability*, *clinical utility*, and *availability* of mobile and wearable devices for monitoring MS, and this is the goal of this study.

Such devices have already been used to monitor the fatigability^5,18^, fatigue^4^, EDSS level^19–22^ and other outcomes of PwMS^23^. For instance, Motl et al. use a two-minute walk test^19^ and the timed 25-foot walk test^20^, which reflect the walking disability level, to approximate the EDSS level. These approaches require the user to manually perform a task and then use the data collected during the task to infer MS symptoms, which might be cumbersome and time-consuming. In addition, they focus only on one aspect of the disease, the motor performance, and neglect the other aspects, which may lead to incomplete approximation. Indeed, a recent analysis of 119 clinical trials related to MS registered in ClinicalTrials.gov shows that 77.31% of the studies focus on motor function tracking and only 5.88% on more novel digital measures (e.g., sleep and cognition tracking)^17^, showing the need for more comprehensive analysis. Other studies use data collected in a specific context, e.g., during the COVID-19 pandemic^23^ or at home^24^, which are difficult to generalize to natural settings. The majority of these studies use simple statistical analysis to show a correlation between disease and objective measures^5,25–27^, which are not meant for automated predictions. Other studies use black-box algorithms^28^, which might be difficult to interpret by patients and clinicians. Considering the complexity of the disease and the heterogeneity of real-world behavior, there is a need for multimodal approaches that consider different aspects of MS.

**Fig. 1:**
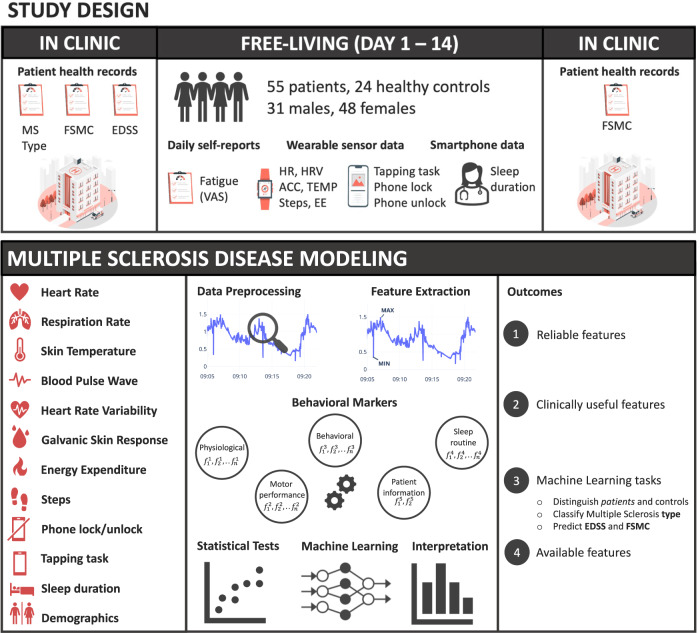
Study design and data modeling setup. We ran the experiments on a dataset we collected from 55 PwMS and 24 healthy controls over two weeks. Our data analysis objectives are to identify the most reliable, clinically useful, and available features derived from mobile and wearable sensor data. Our machine learning pipeline identifies the best-performing features for four tasks 1) *PwMS* and *healthy* controls classification, 2) MS type classification into *none*, *RRMS* and *PPMS-SPMS*, 3) predict the disability and fatigue levels using wearable sensor data, smartphone usage, and demographics. [Illustrations by Storyset (https://storyset.com/)].

In this paper, we investigate whether data from wearable devices and smartphones can be used for monitoring different aspects of MS disease. The objectives of the paper are: 1) to identify the most reliable features derived from mobile and wearable devices for monitoring MS; 2) to explore the clinical utility of the features derived from mobile and wearable data for monitoring MS; 3) to evaluate the feasibility of using machine learning for automatic assessment of clinical measurements; and 4) to investigate the availability of such data in MS population. To achieve these goals, we use statistical analysis and machine learning to analyze passive sensor data collected using wearable devices, motor performance tests collected via smartphones, and patient information obtained from the hospital to distinguish between PwMS and controls as well as to recognize the disease disability and fatigue levels. We refer to all these aspects as *MS modeling*. In collaboration with clinicians and from the MS disease literature, we identify and combine four phenomena observable through mobile and wearable devices, namely, *physiological*, *behavioral*, *motor performance* and *sleep routine*, and use them to model MS. Accordingly, we then identify the sensor types that could be used to reflect these phenomena and derive a set of features from raw sensor data to represent them. In particular, we explore the effectiveness of each phenomenon alone and their combination thereof to accurately model MS. Considering aspects such as physiological conditions, reflected by heart rate variability or skin temperature, and sleep behavior changes in addition to motor performance tests provides a more comprehensive view of the disease. Fig. 1 presents an overview of the study design, data modeling steps, and MS disease aspects investigated in this study.

## Results

### Demographics

Table 1 presents the demographics and disease-related information of subjects in our dataset. The dataset contains data from 79 participants, 31 males, and 48 females with an average age of 34 years. Out of the 79 participants, 55 were PwMS, and 24 were healthy controls. The mean EDSS level was 2.58, with a range of values from 0 to 6. The EDSS scores were measured during two clinic visits, and they were consistent across the two visits.

**Table 1:** Demographics data – Mean (standard deviation) of the demographics of the participants in our dataset

		All	Control	PwMS
Participants		79	24	55
Demographics	Age, mean (std)	35.15 (10.05)	33.5 (10.6)	36.8 (9.5)
	Gender	31 M & 48 F	11 M & 13 F	20 M & 35 F
	Asian	3	3	–
	Caucasian	69	18	51
	Hispanic	1	1	–
	Middle-Eastern	5	2	3
Health Information	EDSS, mean (std)	–	–	2.58 (1.6)
	FSMC, mean (std)	–	–	55.0 (23.2)
MS Type	RRMS	–	–	46 (83.6%)
	PMS	–	–	9 (16.4%)

### Reliability of features derived from mobile and wearable sensors for MS monitoring

We investigate the reliability of features derived from mobile and wearable devices. Reliability, defined as *the extent to which measurements can be replicated*, reflects both the level of correlation and degree of agreement between measurements^29^. To identify the reliable features, we first extracted features from sensor data and then performed the test-retest reliability analysis^30^. The term features refer to statistical features derived from the time and frequency domain of the signals, which we explain in detail in Section *Feature Extraction*. We consider the features with an intraclass correlation coefficient (ICC) of more than or equal to 0.6 as reliable, similar to Woelfle et al.^31^. Table 2 presents a summary of the ICC and confidence intervals (CIs) for both daily and weekly-aggregated features. Overall, 18 out of 47 features extracted and aggregated daily met the ICC criteria. Features aggregated weekly, which refers to two average values per participant over the two weeks, resulted in higher ICCs. The majority of weekly features, 41 out of 47, met the ICC criteria. Features collected daily show higher variability with lower test-retest reliability in comparison to weekly-aggregated features. This is expected because daily features are highly impacted by people’s behavior during the day. For instance, the physical activity of the user during a day might be very different from another day, i.e., a user might go running on Monday and work from home all day on Tuesday. Such behaviors cause a larger difference in the ICC of the mean physical activity between these two days. On the weekly level, however, users might follow the same weekly routine. We believe that this is the reason why the ICC values for some of the features e.g., mean physical activity, have a large difference on the daily and weekly level.

**Table 2:** Reliability and clinical utility of the features derived from mobile and wearable sensors explored in this work

Sensor	Feature	Daily average		Weekly average		EDSS	FSMC	PwMS vs HC
Coefficient		ICC	CI	ICC	CI	CORR	CORR	*P* value
Physical	Max	0.55	[0.19 0.98]	**0.72**	[0.49 0.86]	−0.24*	−0.13	<0.001
Activity	Mean	0.03	[−0.06 0.82]	**0.80**	[0.62 0.9]	−0.35*	−0.32	<0.001
	Min	0.00	[0.1 0.37]	0.01	[−0.37 0.37]	0.18	0.22	>0.01
	Skew	0.17	[−0.01 0.92]	**0.85**	[0.71 0.93]	0.07	0.03	<0.001
	Std	0.02	[−0.06 0.81]	**0.81**	[0.63 0.91]	−0.31	−0.32	<0.001
Steps	Max	0.09	[−0.02 0.87]	0.24	[−0.15 0.56]	−0.04	−0.19	<0.001
	Mean	0.06	[−0.04 0.85]	**0.83**	[0.67 0.92]	−0.41*	−0.28	<0.01
	Skew	0.14	[−0.01 0.91]	0.46	[0.11 0.71]	0.40*	0.20	<0.001
	Std	0.18	[−0. 0.92]	**0.84**	[0.69 0.92]	−0.44**	−0.30	<0.001
	Sum	0.13	[−0.01 0.9]	**0.84**	[0.68 0.92]	−0.40*	−0.28	<0.01
Blood	Max	−0.02	[−0.06 0.6]	0.45	[0.1 0.7]	−0.04	0.10	<0.001
Perfusion	Mean	**0.69**	[0.33 0.99]	**0.76**	[0.55 0.88]	0.11	0.12	>0.01
(BP)	Min	0.00	[0.01, 0.92]	0.00	[0.05, 0.22]	−0.05	0.04	>0.01
	Skew	0.17	[0. 0.91]	**0.57**	[0.27 0.78]	−0.02	0.01	<0.01
	Std	0.26	[0.05 0.94]	**0.67**	[0.4 0.83]	0.15	0.28	<0.001
Blood	Max	−0.02	[−0.03 0.5]	0.35	[0.1 0.6]	−0.18	−0.22	>0.01
Pulse	Mean	−0.03	[−0.05 0.43]	**0.79**	[0.61 0.9]	0.31	0.38*	<0.001
Wave	Min	0.00	[0.02 0.64]	0.02	[−0.05 0.78]	−0.05	0.04	>0.01
(BPW)	Skew	0.53	[0.17 0.98]	**0.85**	[0.71 0.93]	−0.32	−0.24	<0.001
	Std	0.15	[0.01 0.9]	**0.77**	[0.56 0.89]	−0.23	−0.05	>0.01
Heart	Max	0.26	[0.03 0.95]	**0.66**	[0.39 0.83]	−0.41*	−0.48**	<0.001
Rate	Mean	0.23	[0.03 0.93]	**0.79**	[0.61 0.9]	−0.01	0.09	>0.01
(HR)	Min	**0.58**	[0.23 0.98]	**0.69**	[0.45 0.85]	0.33	0.44**	<0.001
	Skew	0.44	[0.12 0.97]	0.37	[0.01 0.65]	−0.00	0.07	>0.01
	std	0.43	[0.11 0.97]	**0.77**	[0.56 0.89]	−0.43**	−0.44**	<0.001
Heart	SD1	**0.88**	[0.64 1.0]	**0.96**	[0.93 0.99]	−0.26	−0.32	<0.001
Rate	SD2	**0.91**	[0.71 1.0]	**0.96**	[0.93 0.99]	−0.38*	−0.47**	<0.001
Variability	nn20	**0.91**	[0.72 1.0]	**0.95**	[0.9 0.98]	−0.46**	−0.50**	<0.001
(HRV)	nn50	**0.91**	[0.74 1.0]	**0.96**	[0.93 0.98]	−0.36*	−0.41*	<0.001
	pnn20	**0.71**	[0.36 0.99]	**0.95**	[0.9 0.98]	−0.44**	−0.48**	<0.001
	pnn50	**0.88**	[0.64 1.0]	**0.96**	[0.93 0.99]	−0.35*	−0.40**	<0.001
	rmssd	**0.88**	[0.64 1.0]	**0.96**	[0.93 0.99]	−0.26	−0.32	<0.001
	sdnn	**0.90**	[0.71 1.0]	**0.97**	[0.94 0.99]	−0.36*	−0.45**	<0.001
	HF	**0.87**	[0.64 1.0]	**0.93**	[0.86 0.97]	−0.16	−0.07	<0.01
	LF	**0.95**	[0.84 1.0]	**0.88**	[0.76 0.94]	−0.14	−0.26	<0.001
	LF/HF	0.23	[0.03 0.94]	**0.95**	[0.91 0.98]	0.29	0.26	<0.001
Skin	Max	0.45	[0.14 0.97]	0.28	[−0.1 0.6]	0.10	0.19	<0.001
Temperature	Mean	0.02	[−0.05 0.78]	**0.89**	[0.79 0.95]	0.26	0.17	<0.001
(TEMP)	Min	0.14	[−0.01 0.9]	**0.96**	[0.92 0.98]	0.12	0.10	<0.001
	Skew	**0.65**	[0.28 0.99]	**0.74**	[0.52 0.87]	−0.05	0.07	>0.01
	Std	0.12	[−0.01 0.89]	**0.87**	[0.75 0.94]	−0.18	−0.07	<0.01
Phone	Locks	**0.89**	[0.68 1.0]	**0.93**	[0.86 0.97]	−0.36*	−0.49**	<0.001
	Unlocks	**0.91**	[0.73 1.0]	**0.91**	[0.73 1.0]	−0.35*	−0.46**	<0.001
Sleep	Hours	0.10	[0.01 0.84]	**0.64**	[0.32 0.82]	0.09	0.06	>0.01
Tapping	Mean tapping frequency (tfm)	0.06	[−0.04 0.84]	**0.71**	[0.47 0.85]	−0.68**	−0.64**	>0.01
task	Tapping count	0.02	[−0.05 0.79]	**0.71**	[0.47 0.86]	−0.65**	−0.62**	>0.01
	Δtfm	0.02	[−0.05 0.79]	**0.74**	[0.5 0.87]	−0.45**	−0.34*	>0.01

ICC refers to the intraclass correlation coefficient, CI to the confidence interval, and CORR to Pearson or Spearman rank correlation. Reliable features with ICC more than or equal to 0.6 are marked in bold. * indicate *p* < 0.01, and ** values indicate *p* < 0.001 with Bonferroni correction. PwMS refers to PwMS and HC refers to healthy controls.

### Clinical utility of features derived from mobile and wearable sensors for MS monitoring

In this section, we explore the clinical utility of the features derived from mobile and wearable sensor data by analyzing their relationship to patient health information (e.g., MS diagnosis, EDSS, and FSMC levels). Table 2 shows a summary of the correlation analysis performed using Pearson correlation or Spearman rank correlation coefficients. We observe a significant correlation between several features derived from sensor data and clinical measures. In particular, we find a significant, negative correlation between EDSS and at least one feature related to participants’ physical activity, number of steps, heart rate (HR), heart rate variability (HRV), phone usage, and performance test. These results indicate that the higher the participants’ EDSS level, the lower their physical activity, variability in the number of steps, heart rate, the number of phone locks/unlocks, and the average tapping frequency. Similarly, at least one feature related to HR, HRV, blood pulse wave (BPW), phone usage, and performance test is significantly correlated to the FSMC. The majority of features show a significant, negative correlation with FSMC. This indicates that the higher the participants’ FSMC level, the lower their average BPW, maximum HR value, HRV, phone usage, and tapping frequency. Supplementary Fig. 1 shows examples of the distribution and correlation between physiological data and clinical measures.

**Fig. 2:**
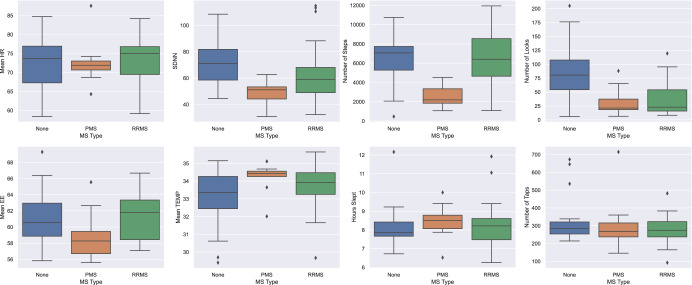
Mobile and wearable sensor data distribution. Distribution of a subset of features for MS Type (*None, RRMS* and *PMS*). The horizontal line within the boxplot displays the median of the data, the box limits refer to the interquartile range (IQR), and the whiskers extend to the minimum and maximum values. The data points falling outside the whiskers are outliers.

We then applied statistical tests to understand the ability of digital features to discriminate between PwMS and healthy controls (HC). The features that were significantly different between the two groups are related to physical activity, number of steps, blood perfusion (BP), BPW, HR, HRV, skin temperature (TEMP), and phone usage. We found no significant difference in sleep duration and performance tests between the two groups. Fig. 2 presents the distribution of some of the features for HC, people with *Relapsing Remitting MS (RRMS)* and people with *Primary or Secondary Progressive MS (PMS)*. We observe higher HRV, shown by the standard deviation of the interbeat intervals feature (SDNN), in HC compared to PwMS (*p* < 0.001 according to Mann-Whitney U test), which is consistent with previous findings that PwMS have a reduced HRV^32^ and HC higher HRV^33,34^. The number of steps feature provides also interesting insights. HC and people with RRMS perform more steps in comparison to people with PMS suggesting that people with PMS are physically less active than the other two groups, confirming previous findings^35^. HC group has overall lower TEMP in comparison to PwMS, which is in line with the findings of Eggenberger et al.^36^ for patients with mild cognitive impairments. We investigated the possibility of this result being confounded with the time of the year the data was collected and did not observe any evidence this might be the case. Overall, these results indicate that the variation in physiological data such as HR, HRV, TEMP, and physical activity levels between PwMS and HC can be observed even during daily activities through wearable technology. These findings serve as a compelling reason for conducting further research in this direction.

### Performance of features derived from mobile and wearable sensor for MS modeling

We developed several models to distinguish between PwMS and HC and to recognize the type of MS a participant is diagnosed with as well as to predict the fatigue severity and the disease disability level. We framed the first two tasks as binary and multi-class classification problems, respectively, and the latter as regression problems. We then trained models in a supervised learning fashion. We used groups of features as input to the explored models. The groups of features we derived are 1) *behavioral* referring to individual’s behavior such as physical activity, and phone usage; 2) *motor performance*, which pertains to the results of the tapping frequency test conducted through a smartphone application; 3) *physiological*, which refer to physiological aspects (e.g., the average value of heart rate); 4) *sleep routine*, which reflects information regarding sleep duration; and 5) *demographics*, including age and gender, described in detail in Section *Methods*. The goal of these experiments is to investigate whether data collected unobtrusively through mobile and wearable devices could complement traditional techniques to distinguish between PwMS and HC as well as to predict disease disability and fatigue levels. For classification tasks, we investigated the performance of shallow classifiers, such as *logistic regression (LR)*, *random forest (RF)*, *extreme gradient boosting (XGB)* and *fully-connected neural network (FCNN)*, and compared them to *random guess (RG)* – which predicts the outcome uniformly at random –, *biased random guess (BRG)* – which predicts the majority class in training set –, and *demographics* – that refers to a classifier with gender and age as features – baselines. For regression problems, we explored the performance of *linear regression (LR)*, *random forest regressor (RFR)*, *extreme gradient boosting regressor (XGBR)*, and *FCNN* with mean squared error (MSE) loss function. We compared the regression results to *random* – that predicts a random number within the expected range – and *average* – that always predicts the average score in the dataset – baselines. To evaluate models’ performance, we calculate the *F1-score (F1)* for classification tasks and *mean absolute error (MAE)* for regression tasks over stratified group 5-fold cross-validation (SG5FCV), similar to^22,23^. We split the data into non-overlapping participant training and test sets to investigate the performance of our approach to a new, unseen group of subjects.

#### PwMS vs HC

**Fig. 3:**
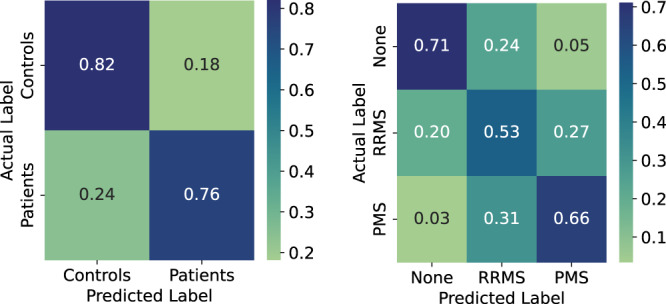
Confusion matrix for classification results. Summed and normalized (per row) confusion matrix of the Stratified Group 5-fold Cross-Validation (left).

Table 3 (left) reports the mean (standard deviation) of the F1-score computed over 50 iterations of the SG5FCV for each classifier. We report the F1-scores for each group of features, namely, *behavioral*, *physiological*, *sleep routine*, and *motor performance* and their combinations. Our results show that using all feature groups explored in this work as input to the classifiers provides the best performance in terms of the F1-score and outperforms the three baselines for distinguishing PwMS and HC. In particular, using the features that represent a participant’s behavior, physiology, sleep routine and motor performance reaches the highest F1-score of 82% to distinguish between PwMS and HC, which is 26, 31, and 16 percentage points higher than BRG, RG, and demographics, respectively. We observe that most of the feature categories alone or combined outperform both baselines. This is however not the case for sleep routine, which seems to not be sufficient for this task. Behavioral features alone or in combination with sleep routine information achieve an F1-score of 78% using the LR classifier, which is 22 and 27 percentage points higher than BRG and RG. These results imply that in case information related to other physiological markers is not available, behavioral markers alone could be used for this task. This is promising because behavioral features can be tracked continuously without effort or discomfort for both patients and clinicians as opposed to, for example, the motor performance tests. Fig. 3 (left) shows the confusion matrix of the best classifier (LR with all feature groups as input) for distinguishing between PwMS and HC. We observe that our approach misclassifies HC only 18% of the time. The majority of the misclassifications occur for MS patients, where in 24% of the cases they are misclassified as HC. This is expected because PwMS in the early stages of the disease can have similar behavior to HC.

**Table 3:** Weighted F1-scores (F1) for each classifier to distinguish PwMS from HC and the MS type (*none*, *RRMS* and *PMS*)

	PwMS vs Healthy (Binary)				MS Type (3-class)			
Group	LR	FCNN	RF	XGB	LR	FCNN	RF	XGB
Behavioral	0.78 (0.10)	0.75 (0.12)	0.72 (0.12)	0.72 (0.11)	**0.62 (0.22)**	0.58 (0.18)	0.55 (0.22)	**0.54 (0.20)**
Physiological	0.73 (0.12)	0.70 (0.12)	0.68 (0.14)	0.68 (0.11)	0.51 (0.15)	0.49 (0.15)	0.47 (0.15)	0.48 (0.15)
Sleep routine	0.37 (0.21)	0.35 (0.20)	0.45 (0.11)	0.46 (0.14)	0.29 (0.12)	0.29 (0.12)	0.30 (0.12)	0.33 (0.12)
Performance	0.76 (0.14)	0.76 (0.15)	0.73 (0.12)	0.70 (0.13)	0.39 (0.13)	0.44 (0.14)	0.44 (0.15)	0.42 (0.15)
Beh+Phys	0.73 (0.12)	0.70 (0.13)	0.69 (0.14)	0.68 (0.12)	0.50 (0.16)	0.48 (0.16)	0.47 (0.15)	0.48 (0.16)
Beh+Sle	0.79 (0.11)	0.74 (0.12)	0.72 (0.12)	0.72 (0.11)	**0.62 (0.22)**	0.59 (0.19)	**0.56 (0.22)**	**0.54 (0.21)**
Beh+Per	0.77 (0.15)	0.77 (0.14)	0.73 (0.12)	0.70 (0.13)	0.40 (0.13)	0.44 (0.14)	0.43 (0.15)	0.41 (0.14)
Phy+Sle	0.73 (0.12)	0.69 (0.13)	0.69 (0.13)	0.69 (0.11)	0.50 (0.16)	0.48 (0.16)	0.45 (0.16)	0.49 (0.16)
Phy+Per	0.74 (0.11)	0.70 (0.12)	0.70 (0.13)	0.67 (0.13)	0.51 (0.16)	0.48 (0.16)	0.44 (0.15)	0.48 (0.16)
Per+Sle	0.76 (0.15)	0.76 (0.15)	0.73 (0.13)	0.70 (0.13)	0.39 (0.13)	0.44 (0.12)	0.44 (0.13)	0.43 (0.14)
Beh+Per+Phy	0.74 (0.12)	0.70 (0.13)	0.69 (0.13)	0.68 (0.11)	0.51 (0.16)	0.47 (0.16)	0.43 (0.15)	0.46 (0.17)
Per+Phy+Sle	0.74 (0.13)	0.70 (0.13)	0.68 (0.13)	0.69 (0.11)	0.50 (0.15)	0.46 (0.15)	0.45 (0.14)	0.46 (0.16)
Beh+Phy+Sle	0.73 (0.12)	0.70 (0.12)	0.68 (0.12)	0.68 (0.11)	0.50 (0.16)	0.48 (0.16)	0.45 (0.16)	0.47 (0.16)
**All**	**0.82 (0.11)**	**0.82 (0.11)**	**0.78 (0.10)**	**0.78 (0.12)**	0.60 (0.18)	**0.60 (0.17)**	0.55 (0.20)	0.51 (0.19)
BRG	0.56 (0.01)				0.36 (0.01)			
RG	0.51 (0.04)				0.34 (0.16)			
Demographics	0.66 (0.12)				0.43 (0.16)			

The classification metrics are reported as mean (standard deviation) of the *stratified group five-fold cross-validation* iterations and 50 runs. The bold values denote the highest F1-score for each classifier.

#### MS type

Table 3 (right) presents the mean (standard deviation) of the F1-score for each classifier to distinguish three classes of MS type. We observe that the majority of classifiers outperform both the RG and BRG baselines, except the ones using only sleep routine or performance features in input. In particular, LR achieves the highest performance with an F1-score of 62%, which is 26, 28, and 19 percentage points higher than the BRG, RG, and demographics. These results demonstrate the capability of our approach to not only provide a high-level classification of individuals into PwMS and HC, but also to distinguish between HC, people with RRMS, and people with PPMS/SPMS. Similar to the previous task, the information related to an individual’s behavior (e.g., physical activity, phone locks/unlocks) plays the most significant role in recognizing MS type. Fig. 3 (right) shows the confusion matrix of the best classifier (FCNN with all feature groups as input) for MS type recognition. We observe that our approach misclassifies HC as RRMS 24% of the time and 5% as PMS. The misclassifications occur mainly between people with PMS and RRMS, which could be due to the few data samples in the PMS group.

#### Disability level

Table 4 (left) reports the MAE for each regressor for predicting the MS disability level measured using EDSS^10^, which is a score from 0 to 6 in our dataset. From the table, we observe that the XGBR achieves the highest performance for predicting the EDSS level, with an MAE of 0.76, which is 0.72, 0.71, and 0.11 points lower than the random, average, and demographics baselines. These results imply that the features related to motor performance alone or in combination with behavioral data or sleep could be used to predict EDSS. These results further confirm the correlation analysis discussed before.

**Table 4:** Average MAE scores for each classifier to distinguish the overall fatigue level measured with *FSMC* (*no fatigue*, *moderate* and *severe*), and MS disability level quantified using *EDSS* (*none, mild* and *severe*)

	EDSS Level – MIN=0, MAX=6, STD=1.46				FSMC Level – MIN=20, MAX=97, STD=20			
Group	LR	FCNN	RFR	XGBR	LR	FCNN	RFR	XGBR
Behavioral	1.56 (0.58)	0.86 (0.12)	0.89 (0.11)	0.94 (0.15)	21.24 (7.87)	26.69 (6.77)	17.66 (3.91)	19.30 (4.27)
Physiological	1.65 (0.29)	1.01 (0.11)	0.96 (0.14)	0.87 (0.09)	30.36 (2.37)	26.12 (6.96)	17.52 (2.55)	19.00 (3.86)
Sleep routine	**0.89 (0.24)**	0.85 (0.21)	0.91 (0.18)	1.03 (0.30)	**15.67 (3.39)**	30.06 (10.1)	**15.25 (2.67)**	17.26 (4.75)
Performance	**0.89 (0.25)**	**0.79 (0.16)**	0.79 (0.22)	**0.76 (0.21)**	20.20 (9.15)	28.38 (10.3)	15.42 (2.96)	**15.13 (3.35)**
Beh+Phy	1.65 (0.29)	1.01 (0.12)	0.96 (0.14)	0.87 (0.09)	30.36 (2.37)	26.12 (6.96)	17.52 (2.50)	19.00 (3.86)
Beh+Sle	1.56 (0.58)	0.86 (0.12)	0.89 (0.10)	0.94 (0.15)	21.24 (7.87)	26.75 (6.84)	17.63 (3.88)	19.30 (4.27)
Beh+Perf	**0.89 (0.25)**	**0.79 (0.16)**	**0.78 (0.22)**	**0.76 (0.21)**	20.20 (9.15)	28.35 (10.2)	15.43 (2.96)	**15.13 (3.35)**
Phy+Sle	1.65 (0.29)	1.02 (0.12)	0.96 (0.14)	0.87 (0.09)	30.36 (2.37)	26.09 (6.97)	17.52 (2.52)	19.00 (3.86)
Phy+Perf	1.65 (0.29)	1.02 (0.13)	0.96 (0.14)	0.87 (0.09)	30.36 (2.37)	26.12 (6.91)	17.53 (2.58)	19.00 (3.86)
Perf+Sle	**0.89 (0.25)**	**0.79 (0.16)**	**0.78 (0.21)**	**0.76 (0.21)**	20.20 (9.15)	28.44 (10.3)	15.45 (3.05)	**15.10 (3.35)**
Beh+Perf+Phy	1.65 (0.29)	1.02 (0.12)	0.96 (0.14)	0.87 (0.09)	30.36 (2.37)	26.10 (6.93)	17.52 (2.55)	19.0 (3.86)
Perf+Phy+Sle	1.65 (0.29)	1.02 (0.12)	0.96 (0.14)	0.87 (0.09)	30.36 (2.37)	26.12 (6.96)	17.53 (2.57)	19.0 (3.86)
Beh+Phy+Sle	1.65 (0.29)	1.01 (0.11)	0.96 (0.14)	0.87 (0.09)	30.36 (2.37)	26.11 (6.95)	17.52 (2.55)	19.0 (3.86)
All	1.59 (0.49)	0.88 (0.12)	0.96 (0.20)	1.03 (0.24)	53.48 (13.3)	**25.81 (6.20)**	17.41 (4.65)	18.0 (5.60)
Random	1.48				30.58			
Average	1.47				26.19			
Demographics	0.87				25.49			

The classification metrics are reported as mean (standard deviation) of the *stratified group five-fold cross-validation* iterations and 50 runs. The bold values denote the lowest MAE score for each regressor.

#### Fatigue level

Table 4 (right) shows the mean (standard deviation) of the MAE for each regressor to predict the level of fatigue measured using FSMC^11^, which is a score from 20 to 97 in our dataset. The XGBR achieves the lowest error with an MAE of 15.13%, which is 15.48, 11.09, and 10.39 percentage points lower than the random, average, and demographics baselines, using the performance test features as input to the classifier. This is expected because performance-related features were among the highly correlated features with FSMC, which were indeed developed to measure motor fatigability as discussed in the previous section.

### Feasibility of collecting mobile and wearable device data from PwMS and HC

**Fig. 4:**
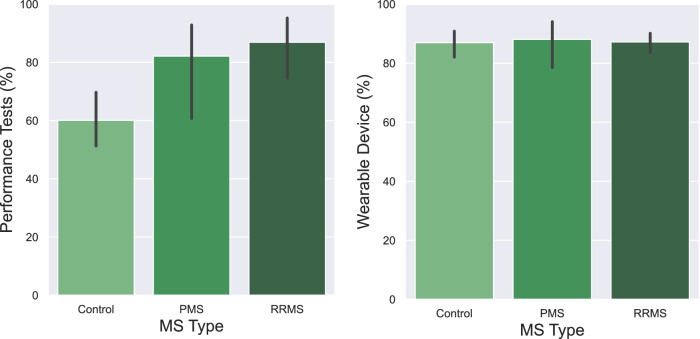
Feasibility to collect smartphone and wearable data. Overview of the amount of data collected with wearable sensors and smartphones (right). The error bars show the difference in the amount of collected data for 14 days.

To evaluate the feasibility of collecting physiological, behavioral, and performance data, we investigated the quantity of collected data for each device type concerning the expected amount of data, similar to Antikainen et al.^34^. Fig. 4 (left) shows the percentage of collected data from the wearable device and smartphone application for each group of participants (e.g., people with PMS, RRMS, or HC). Wearable device data was available for 79 participants, and performance tests for 60 participants. The reduction of the number of participants from 79 to 60 for performance tests is because 19 participants had incomplete data. From the figure, we further observe that the wearable device has a higher compliance rate (on average around 82%) in comparison to performance tests (on average around 74%). These results imply that participants wore the wearable device for the majority of the day. In addition, there is no difference in the compliance rate across groups of participants for wearable devices. However, HC did not complete as many performance tests as PwMS.

## Discussion

Taken together, the results showed that 16 out of 47 daily-aggregated features and 38 out of 47 weekly-aggregated features demonstrated good-to-excellent test-retest reliability. The correlation analysis revealed that a smaller group of features, including at least one feature related to physical activity, number of steps, HR, HRV, phone usage, and tapping task significantly correlated to EDSS and FSMC. Statistical tests demonstrated that several features can discriminate between PwMS and HC. These results indicate the clinical utility of features derived from mobile and wearable sensor data for monitoring MS disability and fatigue aspects as well as distinguishing between PwMS and HC. The machine learning approach achieved an F1-score of 82% in distinguishing between PwMS and HC, 62% in recognizing the MS type, an MAE score of 0.76 in predicting the EDSS levels, and 15.13 in estimating the FSMC level. Study participants showed a higher compliance rate to wearable devices in comparison to smartphone-based performance tests.

Our findings build upon a small body of previous work that explored the feasibility of using wearable sensors and smartphones to distinguish between PwMS and HC as well as to predict MS disease disability and fatigue levels. They highlight the most reliable, clinically useful, and available digital features for MS monitoring. These findings serve as guidelines for both medical researchers and clinicians to identify the type of data to be used for MS monitoring in real-world scenarios.

Several authors investigated the feasibility of using smartphone data to predict the EDSS level^21,22,25,37^. Chitnis et al.^25^, for instance, found significant correlations between several biosensor-derived features with EDSS. We build upon this work and compare the performance of smartphone-based approaches with passive, wearable sensing techniques and find that these behavioral markers perform comparably well to smartphone-based, motor performance tasks. In addition, we investigate the relationship of sensor-derived features to FSMC and their ability to discriminate between PwMS and HC.

Very few studies examined fatigue assessment using smartphone and wearable sensor data^4,38–40^. These approaches focus on healthy individuals (e.g., refs. ^38–40^), which might not generalize to PwMS. Only a few researchers investigated the feasibility of using mobile and wearable sensor data to distinguish between PwMS and HC. Schwab et al.^28^, for instance, use smartphone-based performance tests – that assess cognitive, movement, and finger dexterity – and inertial sensor data to distinguish PwMS from HC. Their approach achieves an F1-score of 80% for recognizing whether a subject is diagnosed with MS using performance-based tests. We build upon this work and show the feasibility of using passively sensed information related to individuals’ physical activity, cardiac activity, and more.

The work most closely related to ours is the one presented by Chikersal et al.^23^. They measure MS symptom burden and binary fatigue (e.g., high or low) using smartphone (e.g., calls, location, and screen activity) and Fitbit (e.g., heart rate, steps, and sleep) data. We extend this work by evaluating the feasibility of MS monitoring in less controlled settings where participants move freely. We further explore the impact of behavioral markers in distinguishing between PwMS and HC, recognizing the three MS subtypes and predicting the MS disability level.

While in most tasks, the performance of classifiers and regressors using objective features are higher than baselines, the combination of three or more feature groups did not lead to better performance for predicting EDSS and FSMC. We believe this behavior is due to the small data size. In addition, the performance of demographics data alone is quite high, indicating that age and gender can effectively be predictors of disease disability and fatigue levels. While demographics can be good indicators, they are not a definitive diagnostic criterion. People of any age can develop MS, and there can be considerable variability in age at diagnosis. In addition, age is static, but digital readouts provide information over longer periods and eventually could be used to spot changes in behavior and their impact on disease progression. Collecting more data and investigating such hypotheses is an interesting direction for future research.

Our findings present exciting possibilities for monitoring MS in real-life situations. These findings have the potential to be used by medical researchers and clinicians for the design and development of wearable-based tools for MS disease monitoring, which have the potential to enhance disease management and monitoring.

## Methods

To investigate the feasibility of using wearable devices and smartphones for MS disease modeling, we use a dataset we collected in natural settings, where participants behaved freely. In this section, we explain the study protocol and participants’ recruitment procedure, the type of collected data, and the data analysis approach we followed. Our method includes steps from conventional statistical analysis and machine learning pipelines such as data cleaning and preprocessing, feature extraction, and classification described as follows.

### Study protocol and participants

We ran a data collection study in November 2019. Fig. 1 provides an overview of the study setup. The study lasted for two weeks and we recruited 55 people diagnosed with multiple sclerosis (MS) and 24 healthy controls. The inclusion criteria for the participants were to own or be willing to use an Android or iOS phone. The exclusion criteria for healthy controls were: being diagnosed with a chronic illness, taking medications regularly, and experiencing fatigue or autonomic dysfunction symptoms. All participants followed the same protocol. The participants were asked to continuously wear the Everion smart armband developed by Biofurmis (previously known as Biovotion) for the whole duration of the study. Also, they used the Querum mobile application to answer surveys and perform tests related to their motor impairment and fatigability. In addition, participants used the app to fill sleep diaries upon waking up and before going to sleep. The surveys were sent daily. Participants were recruited at the University Hospital of Zurich, Switzerland, from the neuroimmunology outpatient clinic. The Cantonal Ethics Committee of Zurich reviewed and approved the study. All participants signed an informed consent form. We removed identifiable information, such as names and contact information, from the collected data before analysis to preserve participants’ privacy and confidentiality.

### Dataset description

The dataset contains *smartphone* data collected using the Querum application, *wearable sensor* data collected using the Everion device, *self-reports* collected via validated questionnaires sent through the mobile application, and *patient health information* obtained from the hospital records. This represents one of the very few datasets available to investigate the problem of continuous MS modeling.

#### Wearable sensor data

**Fig. 5:**
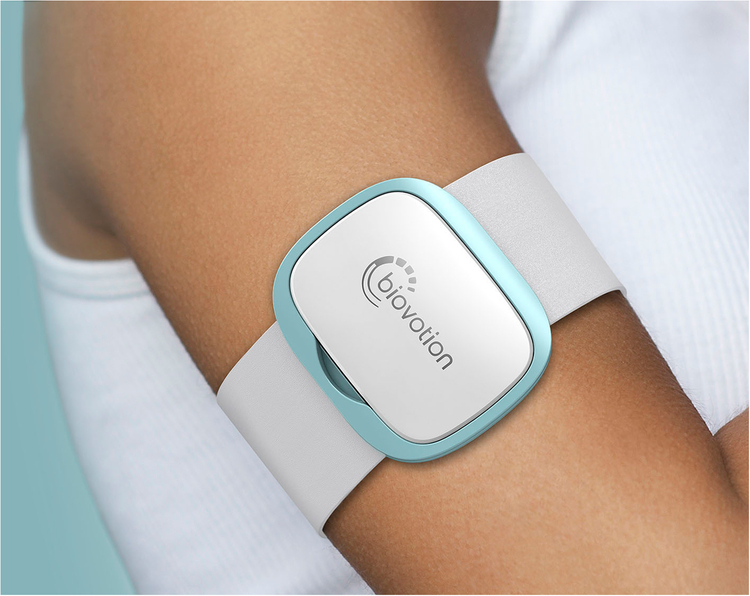
Wearable device. The *Biovotion Everion* wearable device we used to collect physiological and behavioral sensor data over the day. [Image: Infinity Design].

The wearable sensor data was collected using the Biovotion Everion armband (https://biofourmis.com/), which is a medically approved armband, that has shown higher accuracy for heart rate measurement in comparison to other devices^26^. Fig. 5 depicts the Everion wearable device. It contains five sensors, namely, an accelerometer (ACC), barometer (BAR), electrodermal activity (EDA), temperature (TEMP), and photoplethysmography (PPG). In addition, the dataset also contains data streams derived from the raw sensor data (e.g., number of steps). Table 5 describes the data extracted from the sensors of the Everion device, their measurement unit, and the sensor from which the data was derived. In particular, our dataset consists of heart rate (HR), heart rate variability (HRV), respiration rate (RR), electrodermal activity (EDA), skin temperature (TEMP), blood pulse wave (BPW), oxygen saturation (*SpO*_2_), activity class (AC), steps, energy expenditure (EE), barometer (BAR). HR reflects the number of times the heart beats per minute; HRV shows the beat-to-beat variations; EDA the sympathetic nervous system arousal during an activity; RR is the number of breaths a person takes per minute; BPW measures the shape and rhythmicity of the pulse wave generated by the heart contractions which travels through the circulatory system; steps refers to the number of steps per day; EE the amount of energy a user consumes for bodily function (e.g., moving, respiration, digestion); BAR reflects the altitude changes of the user while wearing the device; AC refers to resting, walking, running, cycling and biking. HR, HRV, and EDA provide information about an individual’s physiological arousal and have been widely used to detect people’s cognitive load, affect, and engagement^41^.

**Table 5:** Sensor-derived data – description of the data derived from sensors collected using the Everion wearable device

Sensor	Data Type	Description	Unit
**PPG**	Heart Rate	Number of heartbeats per minute (bpm).	bpm
	Blood Perfusion	Process of a body delivering blood to a capillary bed in its biological tissue.	–
	Blood Pulse Wave	Indicator of shape and rhythmicity of the blood pulse wave.	–
	Heart Rate Variability	Time between heartbeats.	ms
	Respiration Rate	Number of breaths per minute (BPM).	BPM
**ACC**	Steps	Number of steps per day.	steps
	Activity	The intensity of motion.	–
	Energy Expenditure	Amount of energy a person uses to complete bodily functions, from moving, breathing, digestion, and respiration.	kCal
**TEMP**	Skin Temperature	Temperature measured on the surface of the skin.	Degrees Celcius
**BAR**	Barometric pressure	Altitude changes of the user while wearing the device.	Mbar
**EDA**	Electrodermal activity	Describes changes in electrical conductivity of the skin. It is a measure of physiological arousal of the sympathetic nervous system.	kOhm

Activity refers to *resting*, *biking*, *walking*, *running*.

#### Smartphone data

**Fig. 6:**
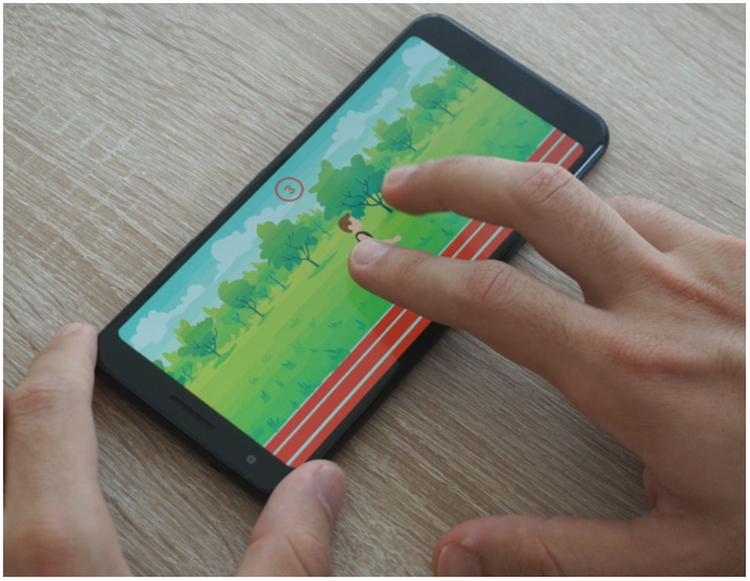
Smartphone application. The *Querum* Android application we used to collect data related to motor performance during the tapping task.

The smartphone data were collected using *Querum* application developed by Barrios et al.^5^. It captures information regarding users’ lock and unlock interactions with the phone, acceleration of the phone, steps and physical activities performed by the user, and places visited. Additionally, participants used the application to complete a finger-tapping task, and the tapping frequency derived from this task has been proposed as an objective measure of fatigue or performance fatigability^5^. Kluger et al.^3^ define fatigability as the “*magnitude or rate of change in a performance criterion relative to a reference value over a given time of task performance*." Fig. 6 shows a screenshot of the Querum mobile application during the tapping task.

#### Patient Health Information

The metadata obtained from the hospital includes demographic information, disease state, and history. In particular, from the hospital, we obtained the gender, age, the MS disability level quantified using the *Expanded Disability Status Scale (EDSS)*, the MS type, when the person had the first symptom and was first diagnosed with MS and disease duration in years. EDSS is a commonly used measure of long-term MS disability as annotated by a clinician. It is a scale that ranges from 0 to 10 with 0.5 increments. Even though MS is a highly heterogeneous and subject-specific disease, PwMS can be grouped into three clinical phenotypes depending on the disease progression, namely, *relapsing-remitting MS (RRMS)*, *secondary progressive MS (SPMS)*, and *primary-progressive MS (PPMS)*^42,43^. RRMS is the most common and initial form of the disease characterized by sudden acute symptoms developing over days before plateauing over weeks or months^44^. RRMS affects the majority (85%) of PwMS.

#### Self-reported data

**Fig. 7:**
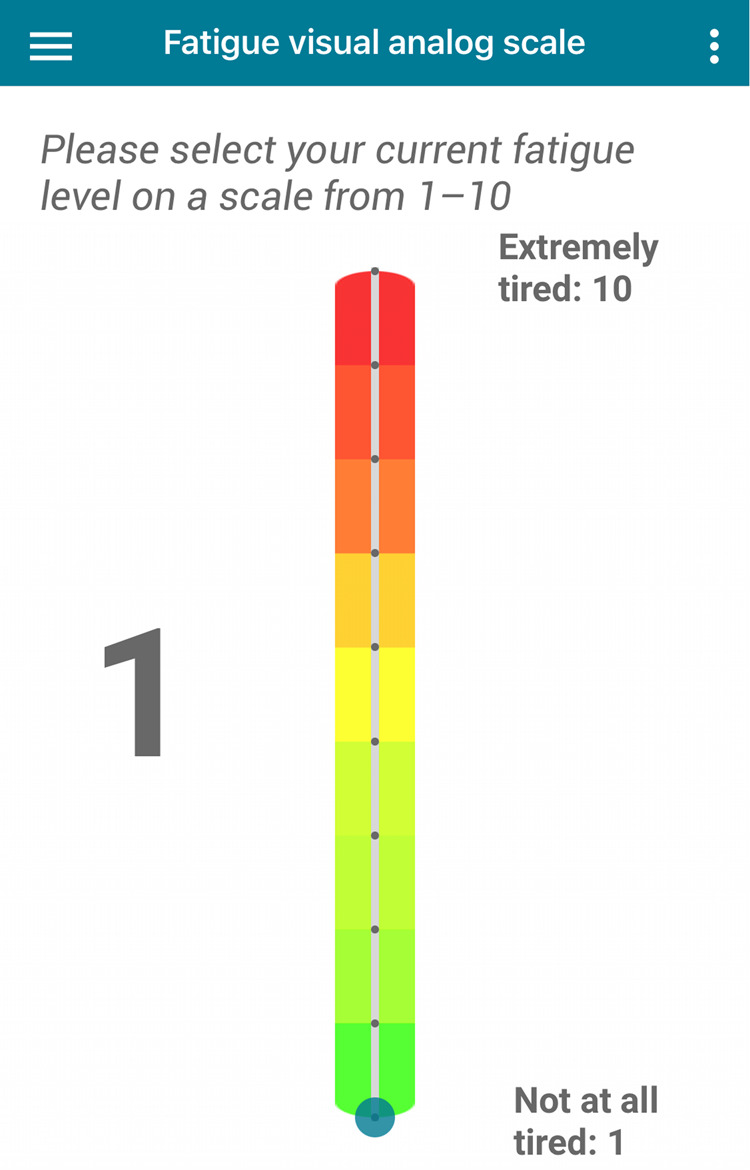
Daily self-reports. The *Visual Analog Scale (VAS)* we used for rating fatigue.

Before and after the study participants completed the *Fatigue Scale for Motor and Cognitive Functions (FSMC)*^11^, Nine-Hole Peg Test (9-HPT)^45^ and handgrip dynamometer. In this study, we used only the FSMC score, which is a validated and reliable measure of cognitive and motor fatigue for PwMS. It consists of 20 items, ten items corresponding to cognitive, and the remaining ten to motor fatigue. Given that the participants completed the FSMC questionnaire before and after the study, we derive a final score as the mean of the two questionnaires. During the study, participants completed daily questionnaires regarding their fatigue level during the day. Participants reported their perceived fatigue level using the Visual Analog Scale (VAS)^12^. VAS was sent three times per day at random times of the day. Participants reported the fatigue level on a scale from 1 (*"Not at all tired"*) to 10 (*"Extremely tired"*) representing how they currently feel. Fig. 7 presents an overview of the VAS questionnaire that was completed by participants on a daily level. The Querum application sent daily notifications to remind the user to complete the questionnaires and the tapping task.

### Data cleaning

With 79 participants enrolled in our study for two weeks, we could expect 1106 days of data available for our analysis. However, after merging the data from all data sources (e.g., smartphone, wearable device), we are left with 60 participants. To prevent discarding data further, we did not consider some data sources for further analysis. In particular, we discarded the steps, acceleration, physical activities, and places collected using the smartphone application because they were present only for a few participants. In addition, the respiration rate and electrodermal activity data collected from the wearable device were of poor quality. For this reason, we did not consider them for further analysis. This amount of discarded data during the data cleaning highlights a general challenge when participants are monitored in real-world settings^46^. Nevertheless, the size of our data set is comparable and in some cases even larger than data sets used in similar studies, e.g., 107 participants for 7 days in Antar et al.^24^, 56 participants for 12 weeks in Chikersal et al.^23^, and 27 participants for 405 days in Luo et al.^38^.

### Data labeling

We use metadata collected by the clinicians to derive labels regarding participants’ MS type, overall disability and fatigue level. These labels were then assigned to the sensor data of each day.

- *PwMS and Healthy Controls:* We define the problem of PwMS and healthy controls recognition as a binary problem. To derive the labels, we divide the participants into *two* groups, PwMS and healthy controls, similar to Schwab et al.^28^. We label all the sensor data of a participant with the corresponding group.
- *MS Type:* We define the problem of MS type recognition as a three-class classification problem. To derive the labels regarding MS type, we divide the participants into *three* groups – *no MS*, *RRMS*, and *PMS* –, consulting with domain experts. The characterization of MS into three groups has also been suggested in the literature^24,27^. There are very few participants diagnosed with SPMS and PPMS, for this reason, we group people with SPMS and PPMS in PMS. Indeed in the MS population, RRMS is the most common subtype is RRMS (85%) and only very few are diagnosed with PMS (15%)^24,47^. Thus, most of the participants in our dataset had RRMS (32) and a few PMS (9), the remaining (19) were 19 healthy controls.
- *Disability Level:* We set up the problem of disease disability level recognition as a regression problem. We derive the MS disability label from the EDSS questionnaire. We assign a 0 EDSS score to the healthy controls.
- *Fatigue Level:* We define the problem of fatigue recognition as a regression task and we label the sensor data of participants using the answers to the FSMC questionnaire. We assign a 0 FSMC score to the healthy controls.

### Feature extraction

Existing measurement methods for fatigue, which is one of the main symptoms of MS, can be grouped in *subjective*, *performance-based*, *sleep routine*, *behavioral* and *physiological*^48^. Following this categorization, we extract 74 features from the wearable and smartphone data, which we divide into four categories and investigate their capability to recognize MS type, disability, and fatigue level. Table 6 presents a detailed overview of the relationship of each feature to MS disease and presents the theoretical foundation of our work. We extracted all the features daily.

**Table 6:** Summary of mobile and wearable sensor data changes for PwMS and healthy controls

Domain	Data stream	Hypothesis for PwMS and healthy controls	Frequency
**Physiological**	Heart Rate (HR)	Increased fatigue is associated with increased HR and reduced HRV^33^.	Continuous
	Heart Rate Variability (HRV)		Continuous
	Blood Pulse Wave (BPW)		Continuous
	Blood Perfusion (BP)		Continuous
	Skin Temperature (ST)	Healthy adults have lower skin temperature than people with mild cognitive impairments^36^.	Continuous
**Behavioral**	Physical Activity	PwMS have problems with balance and feeling dizzy, which can have knock-on effects on their walking. PwMS are less physically active than healthy controls^35^.	Continuous
	Steps	People’s activity influences MS symptoms (e.g., fatigue), which in turn impact the MS disability level^54^.	Continuous
	Phone locks/unlocks	These features could reflect smartphone usage, which might inform the ability to concentrate and the extend of sedentary behavior.	Event-based
**Motor** **performance**	Tapping Task	Decline in performance during a tapping task, or *fatigability*, has been previously shown as a promising objective marker of fatigue^3^.	Daily
**Sleep** **routine**	Sleep Duration	Sleep disturbances are significantly higher in PwMS than in the general population. They may affect women with MS more than men^63^.	Daily
**Patient**	Age	Age is positively related to disease severity^53^ and functioning of PwMS.	Once
**information**	Gender	Females are more prone to MS than males^52^.	Once

#### Motor performance features

Performance-based measurements consist of conducting tests that assess subjects’ motor and cognitive performance on a task^48^. These include tests that quantify walking ability, balance, cognition, and dexterity^21^. Performance on such tests is then used to assess, for instance, subjects’ level of fatigue^5,49^, disability level^21^ and to distinguish between PwMS and HC^28^. Barrios et al.^5^, for example, propose using tapping frequency as an objective smartphone-based measure of self-reported motor fatigability. In a follow-up study, the authors introduced cFAST^49^, a smartphone-based test that quantifies cognitive fatigue. Schwab et al.^28^ use performance-based tests to distinguish between people with and without MS. We build upon this work by further recognizing not only the presence of MS but also the MS sub-type. Roy et al.^21^ suggest using smartphone-based performance measures in conjunction with demographics data. In this work, we use tapping task-related features proposed by Barrios et al.^5^ along with other features to understand its capability for MS modeling. In particular, we extract the number of taps for the whole task (count), the average tapping frequency (tfm), and the difference in the tapping frequency at the beginning and end of the task (Δtfm). We hypothesize that the tapping features in combination with passively collected sensor data will play a discriminative role in recognizing disease disability level and fatigue severity. A limitation of such techniques is however that they require active input from the user, which might be difficult to obtain over long periods in free-living settings.

#### Physiological features

Changes in physiological signals that reflect brain and cardiac activity have been used to detect symptoms of PwMS^27,33^ and other neurodegenerative diseases^36^. For instance, Escorihuela et al.^33^ found that increased fatigue is associated with increased heart rate and reduced heart rate variability. For these reasons, we extract features from physiological signals, which we hypothesize to be indicative of MS outcomes and symptoms. To characterize changes in physiology, we extracted 42 features from the physiological signals collected with the wearable device. In particular, we extracted time-domain statistical features such as minimum, maximum, mean, standard deviation, and skewness from HR, ST, BP, and BPW. To preprocess the PPG signal, we followed procedures suggested in the literature,^27,50^. In particular, we first extracted the inter-beat interval (IBI) from the PPG signal. We then detected and removed artifacts in IBIs and linearly interpolated the missing data, similar to^27^. We then segment the data into 5-minute segments and discard the segments with more than four interpolated IBIs and with excessive movement. From the remaining IBIs, we extracted two groups of features: *time-* and *frequency-domain*. In the time domain, we derive the standard deviation of the IBI (SDNN), the square root of the mean squared differences of successive IBI (RMSSD), the number of interval differences of successive IBI greater than 50 ms (NN50) and 20 ms (NN20) and the proportion derived by dividing NN50 and NN20 by the total number of IBI (pNN50 and pNN20, respectively). Such features capture high-frequency variations in heart rate. To obtain the frequency-domain features, we first apply the Fast Fourier Transform algorithm, then extract the high frequency (HF) and low frequency (LF) as well as the ratio between the two (LF/HF). HF and LF are approximations of parasympathetic and sympathetic activity, respectively.

#### Behavioral features

In healthy controls, behavioral-based methods use behavioral cues – such as yawning, eye closure, sighing – to detect fatigue^48^. In this work, we consider physical activity and the number of steps as behavioral cues of individuals’ MS outcomes and fatigue. This is because it is expected that PwMS move less in general to healthy controls. Dalla et al. 2017^37^ proposed to objectively estimate EDSS using GPS data. Their results show the GPS measurements had a higher level of agreement with neurologists’ reports (intra-class correlation coefficient of 0.68) rather than patients’ (intra-class correlation coefficient of 0.29) showing first the capability of passively sensed data to be used for disease diagnosis. Creagh et al. 2022^22^ used inertial sensor data collected during a two-minute walking test to predict three levels of participants’ EDSS. They used a dataset collected with the Floodlight application from 24 healthy controls, 52 mildly disabled, and 21 moderately disabled patients. Their results show a correlation between the EDSS score and the inertial sensor data collected during the test. Building upon these findings, we investigate whether physical activity-related features could inform the recognition of not only disease disability level, but also distinguish PwMS from the control group and recognize the fatigue severity level. To quantify changes in behavior, we extract features from the physical activity type and intensity as well as the number of steps. In particular, we extract time-domain statistical features (e.g., minimum, maximum, mean, standard deviation, and skewness) from the number of steps and physical activity intensity. We also compute the total number of steps per day. In addition, we compute the total number of locks/unlocks that could inform about the overall interaction with the phone during the day.

#### Sleep routine features

Another type of information that could be indicative of MS symptoms is sleeping behavior, such as sleep and wake-up patterns, circadian cycle, and work-rest patterns^48^. We labeled the sleep and wake events manually by inspecting the movement and HR data similar to Hilty et al.^27^. From the sleep and wake-up time, we then derive the total hours slept and use it as a feature to recognize MS outcomes and fatigue. A limitation of this technique is that it is based on the findings on an overall population level, however, sleep needs and patterns differ among individuals^14,48^.

#### Demographics information

Studies have also shown that demographic information, e.g., age and gender, can be predictive of MS and its symptoms^28,51^. This might be because women are three times more at risk of MS than men^52^. In addition, age is positively related to disease severity^53^. Therefore, we consider gender and age as additional information and use them as features of the model.

#### Patient health information

Research has shown that MS conditions (e.g., MS type) influence symptom changes and the overall functional ability (the inverse of disability) of PwMS^24,53,54^. Thus, we used the MS type (e.g., none, RRMS and PPMS/SPMS) as a feature to predict the disease disability level (EDSS) and fatigue level (FSMC).

### Classification & regression

To investigate whether MS type, disability, severity, and fatigue could be linked to physiological and behavioral data collected from smartphones and wearable sensors, we train machine learning classifiers and regressors on different groups of features. We implement our approach using machine learning classifiers and regressors that allow the interpretation of the model’s predictions. Although representation-based learning techniques that directly model a task from raw time series are increasingly being employed in the medical domain, interpretability of the findings, model diagnostics and complexity remain largely unsolved issues^55^. We also implement a simple version of deep neural networks and compare its results with the other classifiers.

Given a number of feature groups *f*_*i*_ where *i* ∈ {0, 1, 2, 3, 4}, a one-hot encoded representation of participants’ gender *g* ∈ {0, 1} and a scalar value representing participants’ age *a* ∈ *N*, our goal is to train a predictive model *P* that produces a score *y* that indicates the likelihood of the given set of features belonging to 1) a participant with or without MS (*y* ∈ {0, 1}), 2) a participant without MS, or with RRMS or PMS (*y* ∈ {0, 1, 2}), 3) participant’s disability level (*y* ∈ [0, 6]), and 4) participant’s overall fatigue level (*y* ∈ [20, 97]).

As predictive model *P*, we explore logistic regression or linear regression (LR), random forests (RF)^56^, extreme gradient boosting (XGB)^57^ and fully connected neural network (FCNN)^58^. We initialize the classifiers and regressors using the default parameters of the sklearn Python library. To account for class imbalance for classification tasks, in the training set, we applied the random undersampling (RUS) algorithm^59^. Additionally, we scaled the features using the minimum-maximum scaler, as a common procedure in machine learning^58^.

#### Evaluation

To evaluate our approach, we use common validation techniques, metrics, and baselines described as follows.

*Evaluation procedure*. We use *stratified group 5-fold cross-validation* (SG5FCV) to evaluate the performance of our approach for each task. This technique splits the dataset into five folds of approximately equal size, each set containing only the data of a group of subjects while trying to preserve the ratio of different labels in each split. It uses four folds as the train set and the remaining fold as a test set. The split was stratified by preserving the number of samples for each class (e.g., MS type) and grouped by participants to ensure that the data of the same participant is not present both in the train and test sets simultaneously. This technique ensures that each group will appear exactly once in the test set across all the folds, which allows for testing the generalizability of our approach to new users. SG5FCV has also been used in the literature for similar problems^22,23^.

*Evaluation metrics*. To test the ability of the classifiers to distinguish between healthy controls and PwMS as well as to recognize the MS type, we report the *weighted F1-score*, which is the harmonic mean of precision and recall^58^. To evaluate the ability of regressors to predict the disease disability and fatigue levels, we use the mean absolute error (MAE) metric, which is the average absolute difference between the actual and the predicted scores. We compute the above metrics in the left-out fold and aggregate the results across the folds.

*Baselines*. To compare the performance of machine learning classifiers, we implement three baselines including a random guess (RG), and a biased random guess (BRG). RG, irrespective of the input, classifies each data sample as either a positive or negative class uniformly at random. BRG always predicts a constant label for the test data according to the majority class in the training set. In addition, we compare the performance of single data groups (e.g., physiological, behavioral) and their combination to understand the impact of each of the considered groups and their combination. For regression tasks, we use two baselines, *random*, which randomly generate a value from the distribution of disability and fatigue levels and regard it as a predicted value, and *average*, which always predicts the average disability or fatigue score. For both classification and regression tasks, we used a demographics baseline, which uses only demographic data such as age and gender as input to the model.

### Statistical analysis

To evaluate the technical validity of the dataset, we investigated the relationship between physiological signals collected with wearable devices. We computed the Pearson product-moment correlation when the data samples conformed to a Gaussian distribution and Spearman’s rank correlation otherwise, as a common procedure in the literature^60^. To verify whether the data conforms to a Gaussian distribution, we used the Shapiro-Wilk test^61^. We compare the p-values against the corrected threshold of *p* = 0.001, to account for the Bonferroni correction for multiple comparisons^62^. We then investigated the difference in the distribution of sensor data for each population type (e.g., PwMS and healthy controls) using the Mann-Whitney U statistical test. This test is commonly used for independent and non-parametric samples as is the case in our dataset. To evaluate test-retest reliability, we aggregated the features derived from mobile and wearable devices on a daily (up to 14 values per participant) and weekly (two values per participant) basis. We then derived the intraclass correlation coefficient (ICC) of each feature. ICC values range from 0 to 1, with 0 being the lowest reliability and 1 being the highest reliability. We considered reliable the features with an ICC more than or equal to 0.6, similar to Woelfle et al.^31^.

### Reporting summary

Further information on research design is available in the Nature Research Reporting Summary linked to this article.

### Supplementary information

Supplementary Information

Reporting Summary

## Data Availability

The de-identified data is available for download at https://zenodo.org/records/10497826.

## Code availability

The data analysis was carried out in Python using open-source libraries, such as TensorFlow (https://github.com/tensorflow) and scikit-learn (https://scikit-learn.org).
