# Supplementary material for: Modeling multiple sclerosis using mobile and wearable sensor data
Source: NPJ Digit Med. 2024 Mar 11;7:64. doi: 10.1038/s41746-024-01025-8 (PMC10928076; doi:10.1038/s41746-024-01025-8)
Supplement: Supplementary file 1 — Supplementary Information [file 41746_2024_1025_MOESM1_ESM.pdf]

Supplementary Information

PHRT Consortium - MMAI-MS Project

Christian Holz (1, 2), Gunnar Rätsch (1, 2), Fernando Perez-Cruz (1, 2, 5), Andreas Lutterotti (4), Cristóbal Esteban (1), Martina Baumann (1), Shkurta Gashi (1, 2), Neda Davoudi (1, 2), Rita Kuznetsova (1), Liliana Barrios (1), Max Moebus (1), Veronika Kana (4), Marc Hilty (4), Ekaterina Krymova (5), Luis Salamanca (5), Firat Ozdemir (5), Isinsu Katircioglu (5).

(1): Department of Computer Science, ETH Zürich, Universitätstrasse 6, 8092 Zürich, Switzerland.
(2): ETH AI Center, ETH Zürich, OAT X11, Andreasstrasse 5, 8092 Zürich, Switzerland.
(4): Department of Neurology, University Hospital Zürich (USZ), Frauenklinikstrasse 26, 8091, Zürich, Switzerland.
(5): Swiss Data Science Center (SDSC), ETH Zürich & EPFL, Turnerstrasse 1, 8092 Zürich, Switzerland.

Dataset Exploration

Figure 1 shows the distribution and relationship between the standard deviation of the interbeat intervals feature (SDNN), mean skin temperature (TEMP), mean heart rate (HR), number of steps, FSMC, EDSS, and VAS scores. We observe a significant correlation between features derived from sensor data. For instance, mean HRV is negatively correlated with mean HR as expected (r=0.40, $p < 0.001$) and shows a significant, but weak correlation with the number of steps (r=0.22, $p < 0.001$). The VAS, FSMC, and EDSS scores are significantly correlated with each other. In particular, the correlation between EDSS and FSMC scores is strong according to Pearson correlation (r=0.73, $p < 0.001$). Their correlation to VAS is weak (VAS-EDSS r=0.33, $p < 0.001$ and VAS-FSMC r=0.37, $p < 0.001$ according to Pearson Correlation). The positive correlation between these variables implies that the higher the disease disability score, the higher the perceived daily fatigue. Similarly, the higher the fatigue severity level, the higher the perceived daily fatigue.

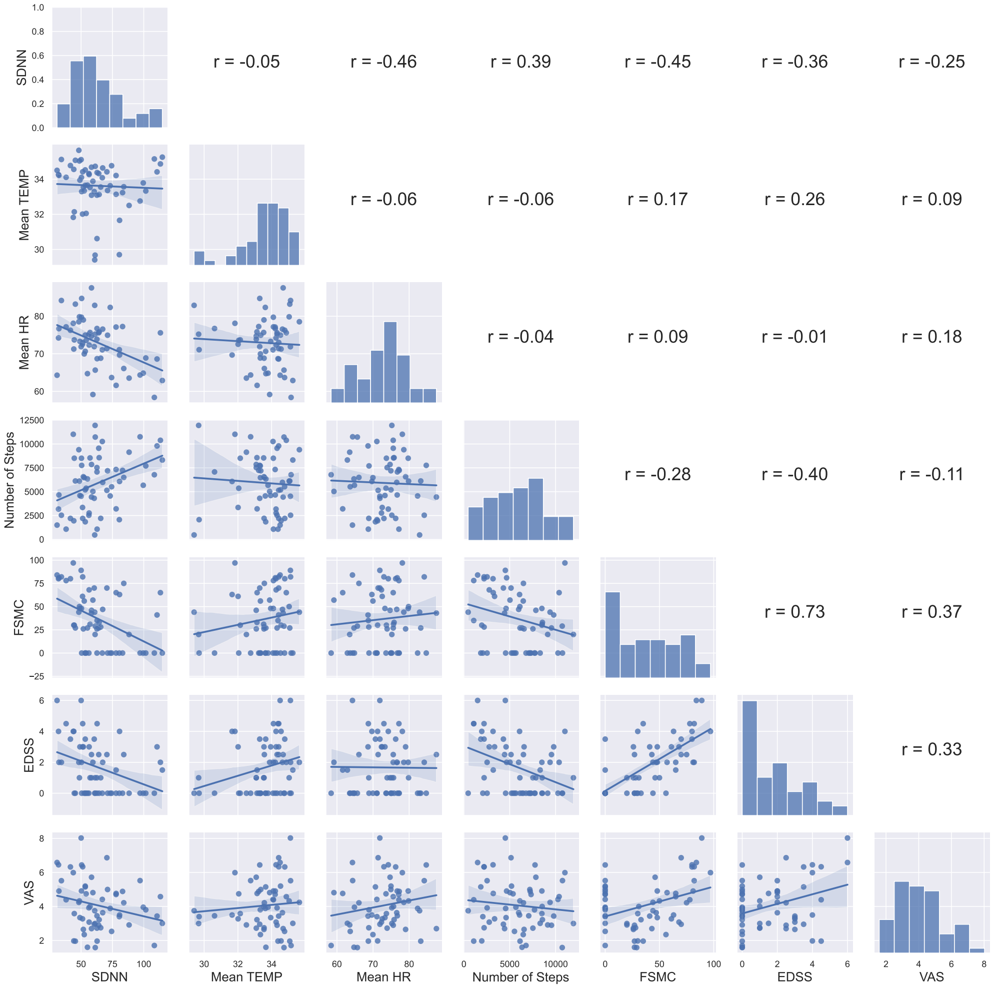

Figure 1. Correlation between features derived from physiological sensors collected with wearable sensors and clinical measures, such as, EDSS, FSMC and VAS.
