# Supplementary material for: Modeling multiple sclerosis using mobile and wearable sensor data
Source: NPJ Digit Med. 2024 Mar 11;7:64. doi: 10.1038/s41746-024-01025-8 (PMC10928076; doi:10.1038/s41746-024-01025-8)
Supplement: Supplementary file 2 — Reporting Summary [file 41746_2024_1025_MOESM2_ESM.pdf]

nature portfolio

Corresponding author(s): Shkurta Gashi (shkurta.gashi@ai.ethz.ch)
Last updated by author(s): Jan 9, 2024

Reporting Summary

Nature Portfolio wishes to improve the reproducibility of the work that we publish. This form provides structure for consistency and transparency in reporting. For further information on Nature Portfolio policies, see our Editorial Policies and the Editorial Policy Checklist.

Statistics

For all statistical analyses, confirm that the following items are present in the figure legend, table legend, main text, or Methods section.

n/a	Confirmed	
☐	☒	The exact sample size (n) for each experimental group/condition, given as a discrete number and unit of measurement
☐	☒	A statement on whether measurements were taken from distinct samples or whether the same sample was measured repeatedly
☐	☒	The statistical test(s) used AND whether they are one- or two-sided<br>*Only common tests should be described solely by name; describe more complex techniques in the Methods section.*
☐	☒	A description of all covariates tested
☐	☒	A description of any assumptions or corrections, such as tests of normality and adjustment for multiple comparisons
☐	☒	A full description of the statistical parameters including central tendency (e.g. means) or other basic estimates (e.g. regression coefficient) AND variation (e.g. standard deviation) or associated estimates of uncertainty (e.g. confidence intervals)
☐	☒	For null hypothesis testing, the test statistic (e.g. F, t, r) with confidence intervals, effect sizes, degrees of freedom and P value noted<br>*Give P values as exact values whenever suitable.*
☐	☒	For Bayesian analysis, information on the choice of priors and Markov chain Monte Carlo settings
☐	☒	For hierarchical and complex designs, identification of the appropriate level for tests and full reporting of outcomes
☐	☒	Estimates of effect sizes (e.g. Cohen's d, Pearson's r), indicating how they were calculated

Our web collection on statistics for biologists contains articles on many of the points above.

Software and code

Policy information about availability of computer code

Data collection	We used Everion arm-worn device for collecting sensor data, a custom-made Android application to collect smartphone data, self-reports and performance tests. We received health related information such as disease duration, disease type from the University of Hospital Zurich (USZ) in an excel sheet.
Data analysis	To analyze the data, we used common packages available in Python such as scipy, numpy, pandas, scikit-learn, pingouin.

For manuscripts utilizing custom algorithms or software that are central to the research but not yet described in published literature, software must be made available to editors and reviewers. We strongly encourage code deposition in a community repository (e.g. GitHub). See the Nature Portfolio guidelines for submitting code & software for further information.

Data

Policy information about availability of data

All manuscripts must include a data availability statement. This statement should provide the following information, where applicable:

- Accession codes, unique identifiers, or web links for publicly available datasets
- A description of any restrictions on data availability
- For clinical datasets or third party data, please ensure that the statement adheres to our policy

We will publish the anonymized data and request for the signature of a data sharing agreement.

Research involving human participants, their data, or biological material

Policy information about studies with human participants or human data. See also policy information about sex, gender (identity/presentation), and sexual orientation and race, ethnicity and racism.

Reporting on sex and gender	We report only information related to gender, which was collected through self-reported surveys.
Reporting on race, ethnicity, or other socially relevant groupings	We report information related to race which was collected through self-reported surveys.
Population characteristics	The dataset contains in total data from 79 participants, 31 males, and 48 females with an average age of 34 years. Out of the 79 participants, 55 were people with MS, and 24 were healthy controls.
Recruitment	Participants were recruited at the University Hospital of Zurich, Switzerland, from the neuroimmunology outpatient clinic.
Ethics oversight	The Cantonal Ethics Committee of Zurich reviewed and approved the study.

Note that full information on the approval of the study protocol must also be provided in the manuscript.

Field-specific reporting

Please select the one below that is the best fit for your research. If you are not sure, read the appropriate sections before making your selection.

☐ Life sciences ☒ Behavioural & social sciences ☐ Ecological, evolutionary & environmental sciences

For a reference copy of the document with all sections, see nature.com/documents/nr-reporting-summary-flat.pdf

Behavioural & social sciences study design

All studies must disclose on these points even when the disclosure is negative.

Study description	The conducted a quantitative research study.
Research sample	The research sample includes 24 healthy controls (11 males and 13 females) with an average age of 33 (standard deviation is 10) and 55 people with multiple sclerosis (20 males and 35 females) with an average age of 36 (standard deviation is 9).
Sampling strategy	We followed a random a convenience sampling strategy and recruited patients from the University Hospital in Zurich.
Data collection	The dataset contains smartphone data collected using the Querum application, wearable sensor data collected using the Everion Device, self-reports collected via validated questionnaires sent through a mobile application, and patient health information obtained from the hospital records.
Timing	We ran the study from November 2019 until June 2020.
Data exclusions	Wearable device data was available for 79 participants, performance tests for 60 participants and self-reports from 94 participants out of 94 recruited participants. The reduction of the number of participants from 94 to 79 is because participants did not comply with the study protocol (N=12), had technical difficulties (N=3), or had incomplete clinical data (N=6).
Non-participation	See above.
Randomization	Participants were allocated to healthy controls or people with multiple sclerosis based on the multiple sclerosis disease diagnosis.

Reporting for specific materials, systems and methods

We require information from authors about some types of materials, experimental systems and methods used in many studies. Here, indicate whether each material, system or method listed is relevant to your study. If you are not sure if a list item applies to your research, read the appropriate section before selecting a response.

Materials & experimental systems

n/a	Involved in the study	
☒	☐	Antibodies
☒	☐	Eukaryotic cell lines
☒	☐	Palaeontology and archaeology
☒	☐	Animals and other organisms
☐	☒	Clinical data
☒	☐	Dual use research of concern
☒	☐	Plants

Methods

n/a	Involved in the study	
☒	☐	ChIP-seq
☒	☐	Flow cytometry
☒	☐	MRI-based neuroimaging

Clinical data

Policy information about clinical studies

All manuscripts should comply with the ICMJE guidelines for publication of clinical research and a completed CONSORT checklist must be included with all submissions.

Clinical trial registration	NA
Study protocol	NA
Data collection	NA
Outcomes	NA

Plants

Seed stocks	NA
Novel plant genotypes	NA
Authentication	NA
